# Evaluation of the predictive and prognostic performance of the PILE score in metastatic renal cell carcinoma

**DOI:** 10.3389/fmed.2026.1835367

**Published:** 2026-07-10

**Authors:** Halil Göksel Güzel, Asım Armağan Aydın, Ayşe Hacıçavuşoğlu, Onur Yazdan Balçık, İsmail Beypınar, Banu Öztürk, Mustafa Yıldız

**Affiliations:** 1 Department of Clinical Oncology, University of Health Sciences, Antalya City Hospital, Antalya, Türkiye; 2 Department of Medical Oncology, University of Medical Sciences, Antalya Training and Research Hospital, Antalya, Türkiye; 3 Department of Internal Medicine, Alaaddin Keykubat University School of Medicine, Alanya Training and Research Hospital, Alanya, Türkiye; 4 Department of Clinical Oncology, Alaaddin Keykubat University School of Medicine, Alanya Training and Research Hospital, Alanya, Türkiye

**Keywords:** PILE score, prognostic biomarker, renal cell carcinoma, treatment response, tyrosine-kinase inhibitor

## Abstract

The PILE score, integrating pan-immune-inflammation value (PIV), lactate dehydrogenase (LDH), and Eastern Cooperative Oncology Group (ECOG) performance status, has been proposed as a composite biomarker in many cancer types. However, its real-world predictive and prognostic performance remains uncertain in metastatic renal cell carcinoma (mRCC). We retrospectively analyzed 67 patients with mRCC treated with first-line tyrosine kinase inhibitor (TKI) monotherapy at two oncology centers. Patients were stratified into low (0–1) and high (≥2) PILE score groups. Patients with low PILE scores demonstrated significantly higher disease control rates compared with those with high PILE scores (90.0% vs. 58.8%, *p* = 0.004), along with a numerically higher objective response rate (68.0% vs. 41.2%, *p* = 0.05), suggesting a potential predictive trend. However, PILE score was not independently associated with progression-free survival (PFS) or overall survival (OS). These findings suggest that the PILE score may function primarily as a predictive marker of treatment response rather than a robust prognostic tool in this setting. Prospective studies are warranted to clarify its potential role in treatment selection.

## 1 Introduction

Renal cell carcinoma (RCC) represents approximately 2–3% of all adult malignancies and is the most common kidney cancer worldwide (1, 2). According to GLOBOCAN 2022, over 430,000 new cases are diagnosed annually, leading to approximately 180,000 deaths per year (3, 4). The prognosis of mRCC remains highly variable, ranging from indolent disease with prolonged stability to rapid progression and poor survival (5, 6). Treatment outcomes have substantially improved with the introduction of vascular endothelial growth factor (VEGF)-targeted tyrosine kinase inhibitors (TKIs) and immune checkpoint inhibitors (ICIs) (7, 8).

Risk stratification in mRCC has traditionally relied on established prognostic scoring systems, most notably the Memorial Sloan Kettering Cancer Center (MSKCC) and International Metastatic RCC Database Consortium (IMDC) models (9). While widely validated and broadly adopted in clinical practice and trial design, these tools incorporate general clinical and biochemical variables and only partially capture the biological complexity of the tumor immune microenvironment (TIME) (5, 6). Over the past decade, hematological indices reflecting systemic inflammation—such as the neutrophil-to-lymphocyte ratio (NLR), systemic immune-inflammation index (SII), systemic inflammation response index (SIRI), and pan-immune-inflammation value (PIV)—have emerged as low-cost, accessible prognostic biomarkers in multiple solid tumors, including RCC (10–13).

Recent studies have specifically examined inflammation-based indices in mRCC patients receiving TKI therapy. In an elderly mRCC cohort treated with first-line VEGF-targeted TKIs, elevated baseline PIV was associated with poorer survival outcomes (14). Similarly, in a multicenter Turkish Oncology Group Kidney Cancer Consortium (TKCC) analysis of patients receiving nivolumab beyond the first line, higher PIV correlated independently with inferior progression-free and overall survival (15). These findings suggest that systemic inflammatory burden may carry prognostic significance across different treatment settings in mRCC, though results remain inconsistent across studies and indices (13).

Despite growing evidence supporting the prognostic utility of individual inflammatory indices in mRCC, no study to date has evaluated the PILE score—a composite index integrating the PIV, Eastern Cooperative Oncology Group (ECOG) performance status, and lactate dehydrogenase (LDH)—in this specific disease setting. This represents a notable gap in the literature, particularly given that the PILE score has demonstrated both prognostic and predictive value in several other advanced cancers treated with immunotherapy and chemo-immunotherapy (16–18).

The PILE score was originally proposed by Guven et al. (16) as a multidimensional prognostic tool combining systemic inflammation (via PIV), host metabolic activity (via LDH), and functional status (via ECOG PS). Given that RCC is a highly vascular and immunogenic tumor, and that VEGF-targeted TKIs directly modulate the tumor’s angiogenic milieu, the biological relevance of a composite biomarker incorporating monocytes—as indirect surrogates of tumor-associated macrophage (TAM) activity—and LDH—a marker of hypoxia-driven metabolic reprogramming—may be particularly pertinent in this setting (19–21). Accordingly, the PILE score may offer a biologically plausible and clinically actionable tool for assessing treatment sensitivity to VEGF-targeted therapies.

Therefore, the aim of this study was to evaluate the predictive and prognostic significance of the PILE score in patients with mRCC receiving first-line TKI monotherapy, and to investigate its potential role as a complementary tool to existing prognostic models.

## 2 Methods

### 2.1 Study population, data collection and clinical follow-up

This retrospective study included 109 patients with histopathologically confirmed renal cell carcinoma who were followed between September 2018 and January 2024 (diagnosis dates ranges between January 2009 to January 2024) at two institutions: the Oncology Clinic of the University of Health Sciences Antalya Training and Research Hospital (UHSATRH) and the Oncology Department of Aladdin Keykubat University Alanya Education and Research Hospital. All patients were stratified according to the IMDC risk classification and subsequently received standard systemic therapy in line with National Comprehensive Cancer Network (NCCN) guidelines.

**Figure 1:**
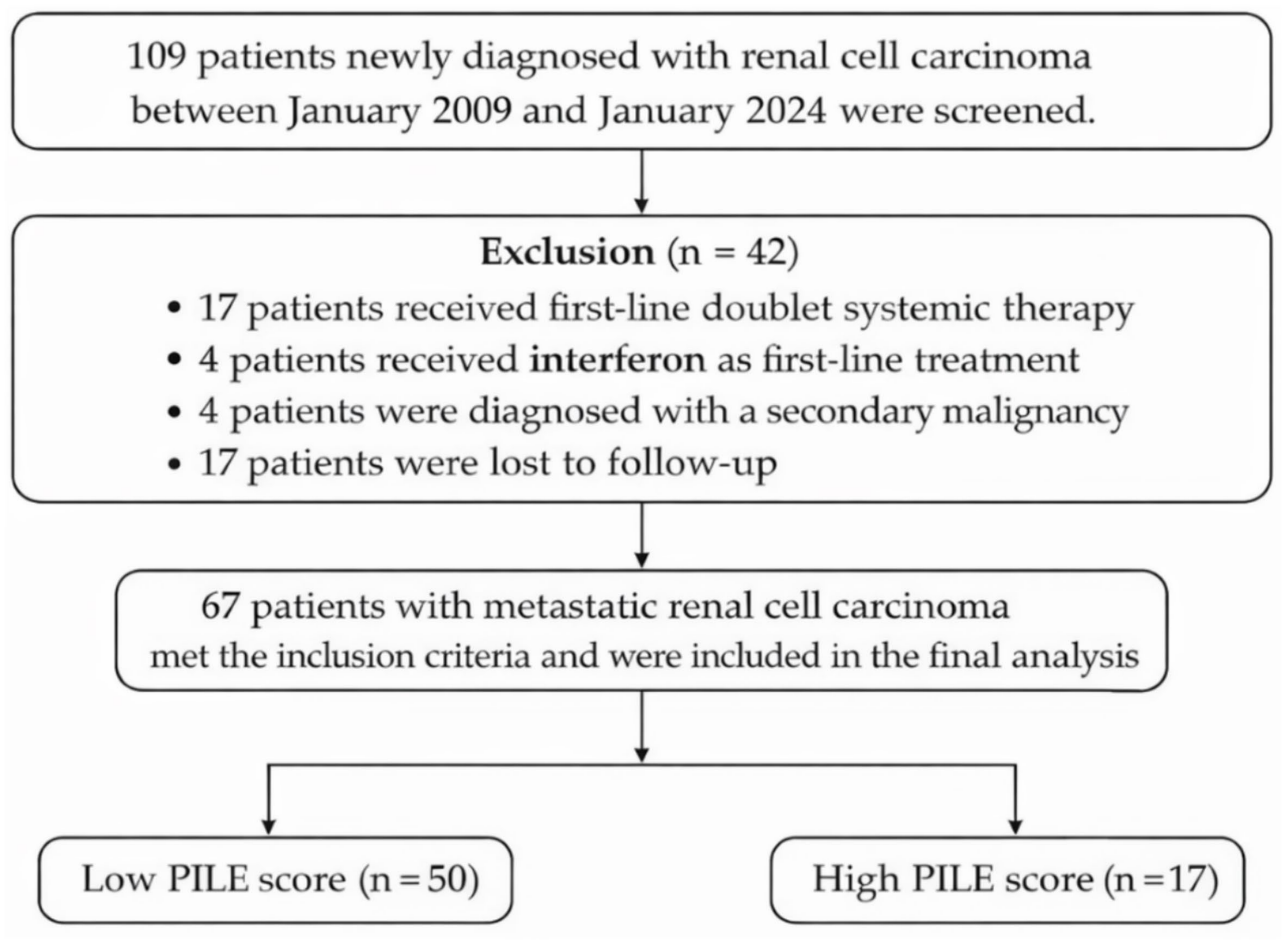
A total of 109 patients with renal cell carcinoma were screened. Patients receiving first-line combination systemic therapy (*n* = 17), interferon therapy (*n* = 4), those with a second primary malignancy (*n* = 4), or those lost to follow-up (*n* = 17) were excluded. The final analysis included 67 patients treated with first-line VEGF-targeted tyrosine kinase inhibitor monotherapy.

The inclusion criteria of the study were a diagnosis of metastatic renal cell carcinoma (RCC), age ≥18 years, histopathological confirmation of RCC, and availability of complete baseline clinical, radiological, and laboratory data at treatment initiation. Exclusion criteria were receipt of first-line combined doublet regimens, including doublet immunotherapy or tyrosine kinase inhibitor plus immunotherapy; treatment with interferon (IFN) as first-line therapy; presence of a second primary malignancy; and loss to follow-up resulting in incomplete clinical data. Accordingly, patients were excluded due to first-line doublet systemic therapy (*n* = 17), first-line IFN treatment (*n* = 4), a second primary malignancy (*n* = 4), or loss to follow-up (*n* = 17). Consequently, as illustrated in the flow diagram, the final analysis included 67 patients with metastatic RCC who met all inclusion criteria and had complete clinical, radiological, and laboratory data available for evaluation (Figure 1).

The following data were collected for all patients from oncology archive files and the hospital electronic medical record system: age, sex, ECOG PS (Eastern Cooperative Oncology Study Group performance status), IMDC risk scores, histological subtypes, tumor grade, presence of sarcomatoid differentiation, history of nephrectomy, sites of metastatic involvement, first-line treatment modality, treatment response, PFS, and OS. Complete blood counts and lactate dehydrogenase (LDH) analyses were performed within 2 weeks prior to the initiation of first-line therapy.

The PILE score, derived from the PIV, LDH, and ECOG performance status, was originally proposed by Guven et al. as a prognostic marker in patients with advanced-stage cancer receiving immunotherapy (16). One point was assigned for each of the following: PIV above the median value, LDH level exceeding the upper limit of normal, or ECOG performance status ≥1. Patients with a total score of 0–1 were classified as having a low PILE score, while those with a score ≥2 were categorized as having a high PILE score. The PIV was calculated using the formula defined by Fucà et al. (22): PIV = (neutrophils × platelets × monocytes)/lymphocytes. For comparative purposes, other widely used indices were also calculated: neutrophil to lymphocyte ratio (NLR) = neutrophils/lymphocytes (13) [NO_PRINTED_FORM], and systemic inflammation response index (SIRI) = (neutrophil count × monocyte count)/lymphocyte count (23).

This study was conducted in compliance with the Declaration of Helsinki (first adopted in 1964 and most recently revised in 2024), with strict observance of its ethical principles. The protocol received full approval from the Institutional Review Board of UHSATRH (Approval Number: 2024–265, 12/20). Given the retrospective design, the requirement for informed consent was waived; nevertheless, all data were anonymized to safeguard patient confidentiality.

### 2.2 Study endpoints and definitions

Patients were staged according to the American Joint Committee on Cancer (AJCC) 7th edition before 2017 while AJCC 8th edition was used for those treated after 2017. Treatment was planned in accordance with NCCN guidelines for the patients. Tumor response was assessed using Response Evaluation Criteria in Solid Tumors (RECIST) v1.1 as a part of routine clinical practice. PFS was defined as the time from the first-line treatment initiation to progression, death, or last follow-up, and OS as the time from the first-line treatment initiation to death from any cause or last follow-up. Tumor response was assessed according to RECIST v1.1. Objective response rate (ORR) was defined as the proportion of patients achieving a complete response (CR) or partial response (PR). Disease control rate (DCR) was defined as the proportion of patients achieving CR, PR, or stable disease (SD).

The primary endpoints of this study were PFS and overall survival OS. Secondary endpoints included ORR and DCR.

### 2.3 Statistical analysis

All statistical analyses were performed using SPSS for Windows, version 22.0 (IBM Corp., Armonk, NY, USA). Categorical variables were summarized as counts and percentages, and continuous variables were presented as medians with minimum and maximum values in brackets (min-max). The patient population was dichotomized to facilitate survival analyses. Initially, patients were categorized into high and low PILE score groups. For NLR, SIRI, and PIV, optimal cut-off values for dichotomization were determined using receiver operating characteristic (ROC) curve analysis (24). ROC analyses were performed for each variable based on 1-year progression-free survival (PFS) to identify the most appropriate cut-off points. According to these cut-off values, patients were subsequently dichotomized into low and high NLR, SIRI, and PIV groups (Supplementary Figure S1). Response rate comparison analyses were performed using chi-square test. Kaplan–Meier survival analysis was applied for univariate survival analyses, while multivariate analyses were conducted using the Cox proportional hazards regression model. Hazard ratios (HRs) and corresponding 95% confidence intervals (CIs) were calculated using Cox regression. Variables with potential clinical relevance were initially selected for inclusion in the multivariate analysis based on a *p* value <0.2 in univariate analyses. However, to maintain the independence assumption of the Cox proportional hazards model and to prevent severe multicollinearity, inflammatory indices derived from overlapping baseline hematological parameters (specifically neutrophils and lymphocytes)—namely NLR, SIRI, and PIV—were excluded from the final multivariate model. Given the limited sample size and event rate, only the PILE score was retained as the primary composite inflammatory index in the multivariate analysis to ensure a robust and reliable assessment of its independent prognostic value alongside other clinicopathological variables. A *p* value <0.05 was considered statistically significant. Due to the standardized treatment protocols based on RCC treatment guidelines across both participating centers and the high degree of homogeneity in patient demographics, no formal adjustment or stratification for center effects was performed in the multivariate models.

## 3 Results

### 3.1 Clinicopathological characteristics of the study population

A total of 67 patients were included in the final analysis (*n* = 67). Median age was 62 (37–87) and 17 (25.4%) were female. Fifty-one patients (76.1%) had clear cell RCC diagnosis. Demographic and clinicopathological characteristics of the study population are presented in Table 1.

**Table 1:** Clinicopathological characteristics of the study population (*n* = 67).

	*n*	%
Age		
<65	44	65.7
≥ 65	23	34.3
Sex		
Female	17	25.4
Male	50	74.6
ECOG PS		
0–1	52	77.6
≥2	15	22.4
Nephrectomy history		
No	14	29.9
Partial or radical nephrectomy	53	70.1
Histology		
Clear cell	51	76.1
Non-clear cell	16	23.9
IMDC risk score		
Good risk	18	26.9
Intermediate-poor risk	49	73.1
Metastatic sites		
Distant lymph node	40	59.7
Lung	40	59.7
Bone	25	37.3
Liver	8	11.9
Brain	2	3.0
First-line therapy		
Sunitinib	45	67.2
Pazopanib	14	20.9
Cabozantinib	8	11.9

ECOG PS, Eastern Cooperative Oncology Study Group Performance Status; IMDC, International Metastatic renal cell carcinoma Database Consortium.

### 3.2 Cut-off points for prognostic inflammatory indices and dichotomisation

A total of 17 patients (25.4%) were categorized as having high PILE scores (2–3). Using the ROC-derived cut-off values 34 patients (50.7%) were classified as having a high NLR (≥2.209), 34 patients (50.7%) had high SIRI value and 34 patients (50.7%) were assigned to the high PIV group (≥390.95) (Table 2).

**Table 2:** Cut-off points for prognostic inflammatory biomarkers.

	*n*	%
NLR (Cut-off: 2.21)		
Low	33	49.3
High	34	50.7
SIRI (Cut-off: 1.36)		
Low	33	49.3
High	34	50.7
PIV (Cut-off: 390.95)		
Low	33	49.3
High	34	50.7
PILE score		
Low (0–1)	50	74.6
High (2–3)	17	25.4

NLR, Neutrophil to lymphocyte ratio; PIV, Pan-immune inflammatory value; ROC, Receiver operating characteristic; SIRI, Systemic inflammation response index.

### 3.3 Response rates according to prognostic inflammatory indices

**Figure 2:**
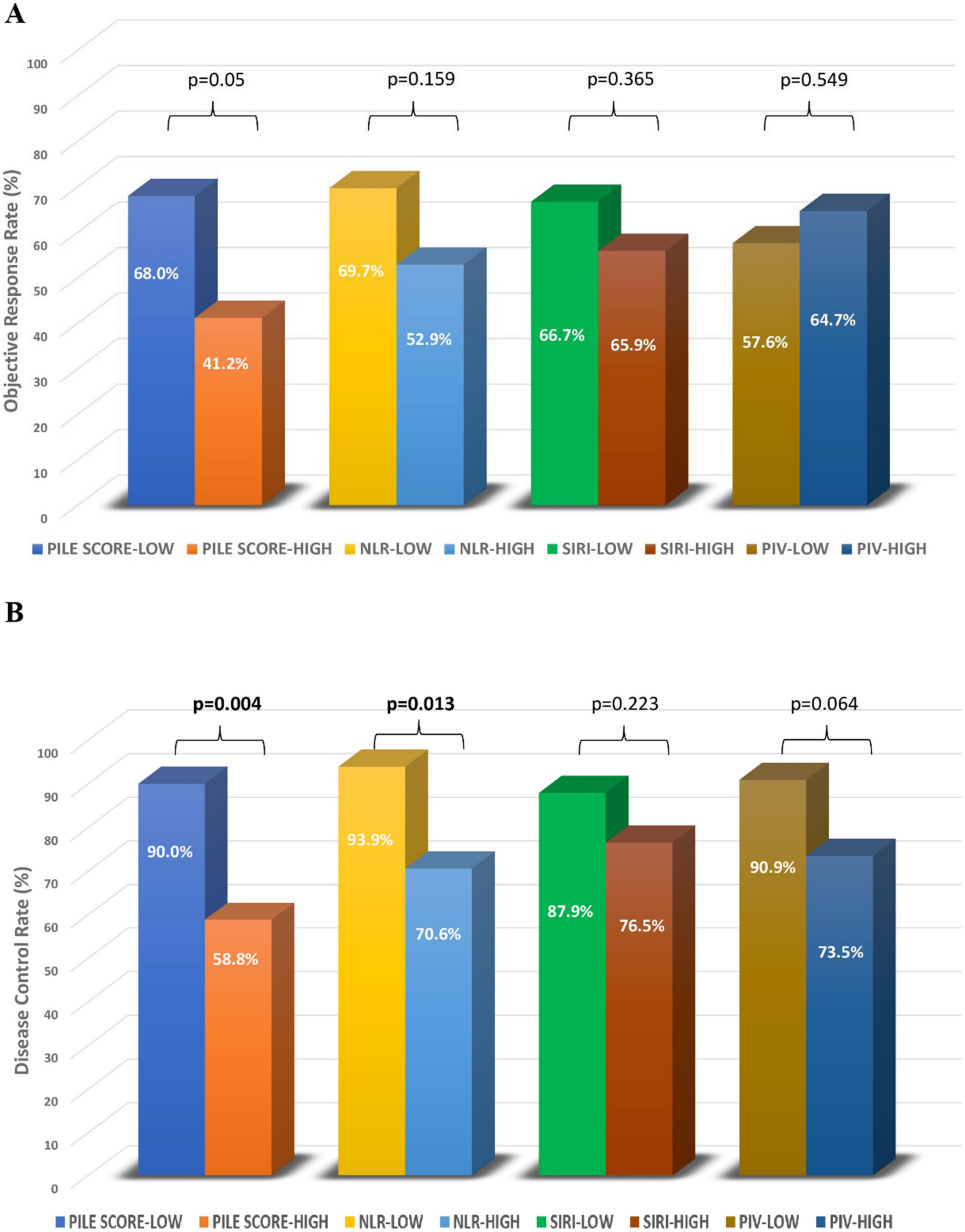
**(A)** Patients with a low PILE score demonstrated a numerically higher objective response rate (ORR) compared with those with a high PILE score (68.0% vs. 41.2%), reaching the threshold of statistical significance (*p* = 0.05), whereas ORR did not differ significantly according to Neutrophil to Lymphocyte Ratio (NLR), Systemic Inflammation Response Index (SIRI), or Pan-immune Inflammation Value (PIV) categories. **(B)** Disease control rate (DCR) according to PILE score and inflammatory indices. A significantly higher DCR was observed in patients with a low PILE score compared with those with a high PILE score (90.0% vs. 58.8%, *p* = 0.004). Similarly, low NLR was associated with improved DCR (*p* = 0.013), while no statistically significant differences were detected for SIRI or PIV.

ORR were analyzed according to PILE score and ROC-derived inflammatory indices. Patients with low PILE score exhibited a higher ORR compared with those with high PILE score (68.0% vs. 41.2%), with the difference reaching the threshold of statistical significance (*p* = 0.05). DCR was significantly higher in patients with low PILE scores compared with those with high PILE scores (90.0% vs. 58.8%, *p* = 0.004). Likewise, patients in the low NLR group demonstrated a significantly improved DCR relative to the high NLR group (93.9% vs. 70.6%, *p* = 0.013) (Figure 2B).

### 3.4 Univariate and multivariate analysis of progression-free survival

**Figure 3:**
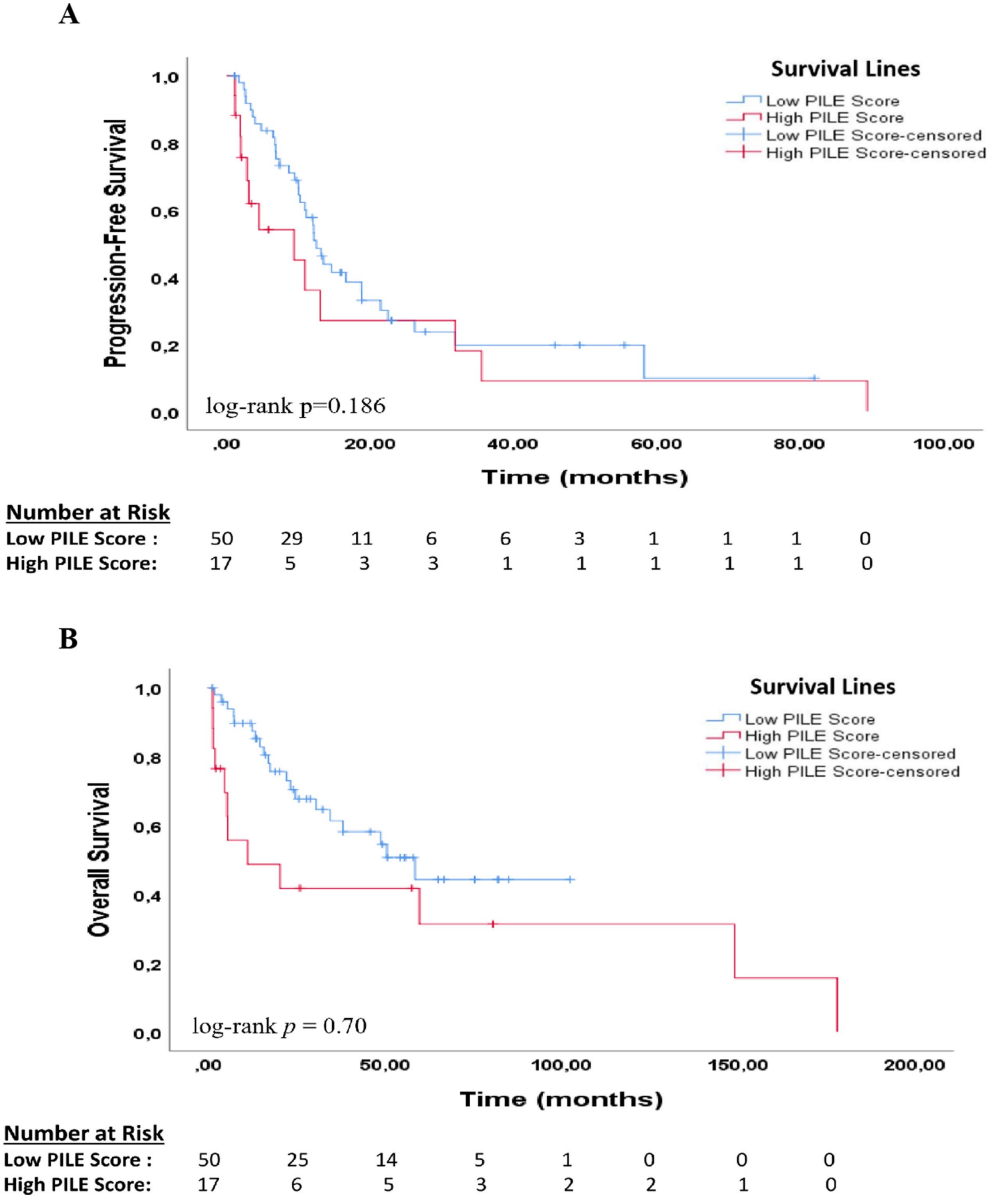
**(A)** Progression-free survival (PFS) stratified by low (0–1) versus high (2–3) PILE score. Patients with a low PILE score exhibited numerically longer median PFS compared with those with a high PILE score [12.5 months (95% CI: 10.7–14.3) vs. 9.4 months (95% CI: 1.3–17.5), respectively, log-rank *p* = 0.186]. **(B)** Overall survival (OS) stratified by PILE score. Although median OS appeared longer in the low PILE score group, no statistically significant difference in OS was observed between groups (log-rank *p* = 0.70).

Patients with a low PILE score demonstrated numerically longer median PFS compared with those with a high PILE score [12.5 months (95% CI: 10.7–14.3) vs. 9.4 months (95% CI: 1.3–17.5), respectively, log-rank *p* = 0.186] (Figure 3A) Similarly, inflammatory indices including high NLR, SIRI, and PIV showed trends toward worse PFS in univariate analysis, but none met the threshold for statistical significance. In the multivariate analysis, non–clear cell histology emerged as the only independent predictor of inferior PFS (HR 3.14, 95% CI 1.44–6.86; *p* = 0.004). In contrast, PILE score did not retain prognostic significance after adjustment for confounding factors (HR 1.62, 95% CI 0.81–3.25; *p* = 0.174) (Table 3).

**Table 3:** Univariate and multivariate analysis of progression-free survival.

	Univariate analysis		Multivariate analysis	
	HR (95% CI)	*p* value	HR (95% CI)	*p* value
Age		0.215		
<65	Reference			
≥ 65	1.45 (0.81–2.60)			
Histology		0.063		0.004
Clear Cell	Reference		Reference	
Non-Clear Cell	1.88 (0.97–3.64)		3.14 (1.44–6.86)	
IMDC Risk		0.217		0.056
Good	Reference		Reference	
Intermediate-Poor	1.56 (0.77–3.14)		2.22 (0.98–5.01)	
First Line Therapy				
Sunitinib	Reference		Reference	
Pazopanib	1.70 (0.86–3.38)	0.129	1.75 (0.88–3.49)	0.114
Cabozantinib	1.34 (0.47–3.87)	0.586	1.64 (0.55–4.84)	0.374
PILE Score		0.186		0.174
Low	Reference		Reference	
High	1.52 (0.80–3.03)		1.62 (0.81–3.25)	
NLR		0.080		
Low	Reference			
High	1.68 (0.94–3.00)			
SIRI		0.063		
Low	Reference			
High	1.75 (0.97–3.15)			
PIV		0.087		
Low	Reference			
High	1.66 (0.92–2.98)			

CI, Confidence interval; HR, Hazard ratio; IMDC, International Metastatic renal cell carcinoma Database Consortium; NLR, Neutrophil to lymphocyte ratio; PIV, Pan-immune inflammatory value; SIRI, Systemic inflammation response index.

### 3.5 Univariate and multivariate analysis of overall survival

The median OS differed numerically but not statistically between patients with low and high PILE score [58.5 months (95% CI: 30.7–86.2) vs. 11.0 months (95% CI: 0.0–38.0), log-rank, *p* = 0.70] (Figure 3B) However, high NLR was significantly associated with inferior OS in univariate analysis (HR 2.32, 95% CI 1.08–4.96; *p* = 0.030). In the multivariate analysis, non–clear cell histology emerged as the sole independent predictor of poorer OS, conferring a more than sevenfold increased risk of mortality compared with clear cell histology (HR 6.16, 95% CI 2.59–14.65; *p* < 0.001) (Table 4).

**Table 4:** Univariate and multivariate analysis of overall survival.

	Univariate analysis		Multivariate analysis	
	HR (95% CI)	*p* value	HR (95% CI)	*p* value
Age		0.245		
<65	Reference			
≥ 65	1.54 (0.75–3.17)			
Histology		0.001		<0.001
Clear Cell	Reference		Reference	
Non-Clear Cell	3.78 (1.76–8.12)		6.16 (2.59–14.65)	
IMDC Risk		0.296		0.060
Good	Reference		Reference	
Intermediate-Poor	1.61 (0.66–3.95)		2.58 (0.96–6.94)	
First line therapy				
Sunitinib	Reference			
Pazopanib	1.04 (0.44–2.43)	0.937		
Cabozantinib	0.53 (0.70–3.95)	0.531		
PILE Score		0.070		0.063
Low	Reference		Reference	
High	2.01 (0.93–4.32)		0.46 (0.20–1.04)	
NLR		0.030		
Low	Reference			
High	2.32 (1.08–4.96)			
SIRI		0.366		
Low	Reference			
High	1.39 (0.68–2.82)			
PIV		0.078		
Low	Reference			
High	1.95 (0.93–4.10)			

CI, Confidence interval; HR, Hazard ratio; IMDC, International Metastatic renal cell carcinoma Database Consortium; NLR, Neutrophil to lymphocyte ratio; PIV, Pan-immune inflammatory value; SIRI, Systemic inflammation response index.

## 4 Discussion

In this two-center retrospective study, we demonstrated that a low PILE score was associated with significantly improved disease control rates while the difference in ORR reached the threshold of statistical significance nature (*p* = 0.05). However, it did not confer an independent prognostic advantage for PFS or OS in multivariate models. Similarly NLR, SIRI, and PIV failed to demonstrate consistent independent prognostic significance after adjustment for clinicopathological variables. In contrast, non-clear cell histology emerged as the dominant determinant of both PFS and OS, highlighting the primacy of intrinsic tumor biology over systemic inflammatory markers in this real-world cohort.

Inflammation-based composite indices have gained considerable attention as low-cost, easily accessible prognostic tools across multiple solid tumors. Building upon this concept, the PILE score was developed by incorporating PIV with lactate dehydrogenase and ECOG performance status, thereby integrating systemic inflammation, tumor metabolic activity, and host functional reserve into a single composite score (16). Early reports, particularly in patients receiving immune checkpoint inhibitors or chemotherapy–immunotherapy combinations, suggested that PILE may have both prognostic and predictive utility. However, accumulating evidence indicates that the performance of such composite indices is highly dependent on tumor type, treatment modality, and disease context, with inconsistent results reported across different cancer subgroups (18).

Although no study has directly investigated the prognostic impact of the PILE score in patients with metastatic renal cell carcinoma, its key inflammatory component, the pan-immune-inflammation value (PIV), has been evaluated in RCC-specific cohorts. In a multicenter analysis conducted by the Turkish Oncology Group Kidney Cancer Consortium, higher baseline PIV was independently associated with inferior progression-free and overall survival in patients with metastatic RCC treated with nivolumab beyond the first line, suggesting that systemic immune-inflammatory burden may influence outcomes in the immunotherapy setting (15). Another point of view for our findings, the absence of independent survival associations should not be interpreted as a lack of biological relevance. The observed association between low PILE score and improved disease control suggests that PILE may be more closely linked to early treatment sensitivity rather than long-term survival outcomes. This observation is consistent with emerging evidence suggesting that systemic inflammation-based indices may preferentially function as predictive markers of treatment response or resistance, rather than as pure prognostic tools independent of therapy, particularly in the context of modern systemic treatments. This hypothesis was supported with previous data showing that systemic inflammation-based indices may better reflect tumor response rates rather than survival outcomes (13, 25). The molecular basis of the predictive role of PILE score in patients who were treated with anti-angiogenic TKIs may be associated with monocytes used in its formulation which was indirectly associated with angiogenesis (19, 20). Moreover, elevated LDH levels which is another component of PILE score, reflect hypoxia-driven metabolic reprogramming and VEGF-dependent angiogenic signaling (21). These data are in line with our observations indicating that the PILE score shows a distinct pattern compared with other inflammatory prognostic indices regarding response rates.

The lack of independent prognostic significance for IMDC risk group in our multivariate model is a notable finding that warrants critical appraisal. One plausible explanation is that non-clear cell histology, which emerged as the dominant prognostic determinant, may itself capture much of the prognostic information encoded in IMDC risk criteria—particularly given the known overlap between non-clear cell subtypes and adverse IMDC features. Additionally, the relatively small sample size and the preponderance of intermediate-poor risk patients (73.1%) in our cohort may have limited the discriminatory power of IMDC risk stratification in this analysis. These observations are consistent with emerging literature suggesting that histological subtype may exert a prognostic influence that supersedes conventional risk model stratification in certain real-world populations (25).

Our study had several limitations. First, the retrospective design introduces inherent selection bias. Second, the relatively small sample size reduces statistical power and may obscure modest associations. Third, the exclusive inclusion of patients treated with first-line VEGF-TKI monotherapy limits extrapolation to contemporary ICI-based combination regimens, where immune-related biomarkers may behave differently. Fourth, inflammatory indices were assessed only at baseline; dynamic changes during treatment, which may better reflect evolving tumor–host interactions, were not captured. Furthermore, as this was an exploratory analysis involving multiple inflammatory indices, no statistical correction for multiple comparisons was performed, which may limit the definitiveness of the marginal *p* values observed in certain parameters like ORR. However, this study was the first in literature aiming to investigate the association of PILE score in patients diagnosed with RCC.

## 5 Conclusion

In metastatic RCC, despite profound changes in therapeutic strategies over the past decade, from mono-TKIs and to immune checkpoint inhibitor based combinations, prognostic stratification has remained largely reliant on historical models such as IMDC (9). Although widely validated, these models are increasingly criticized for their limited ability to reflect tumor immune microenvironment dynamics and molecular heterogeneity, particularly in the immunotherapy era. Nevertheless, no alternative biomarker or composite index has yet demonstrated sufficient robustness, reproducibility, and incremental clinical value to replace IMDC in routine practice (5). Our findings suggest that while the PILE score may not offer incremental prognostic value beyond the established IMDC model, it holds potential as a complementary predictive tool for assessing treatment response in patients with metastatic renal cell carcinoma receiving first-line tyrosine kinase inhibitors. These findings should be considered preliminary and suggestive of a potential predictive trend rather than a definitive advantage. Pending validation in larger, prospective cohorts, the PILE score may offer supplementary utility in evaluating treatment sensitivity for patients receiving first-line tyrosine kinase inhibitors. If confirmed, this index could contribute to the clinical assessment process when selecting between TKI-based regimens and TKI-free therapies in the management of metastatic RCC.

## Data Availability

The raw data supporting the conclusions of this article will be made available by the authors, without undue reservation.

## Supplementary material

SUPPLEMENTARY FİGURE S1 ROC curve analysis of **(A)** Neutrophil to Lymphocyte Ratio (NLR), **(B)** Systemic Inflammation Response Index (SIRI), and **(C)** Pan-immune Inflammation Value (PIV). The table summarizes the diagnostic performance, including Area Under Curve (AUC), 95% Confidence Interval (CI), and *p*-values. All inflammatory indices demonstrated non-significant predictive value (*p* > 0.05).
